# Development of grouped icEEG for the study of cognitive processing

**DOI:** 10.3389/fpsyg.2015.01008

**Published:** 2015-07-21

**Authors:** Cihan M. Kadipasaoglu, Kiefer Forseth, Meagan Whaley, Christopher R. Conner, Matthew J. Rollo, Vatche G. Baboyan, Nitin Tandon

**Affiliations:** ^1^Vivian Smith Department of Neurosurgery, University of Texas Health Science Center at HoustonHouston, TX, USA; ^2^Department of Computational and Applied Mathematics, Rice UniversityHouston, TX, USA; ^3^Texas Medical Center, Mischer Neuroscience Institute, Memorial Hermann HospitalHouston, TX, USA

**Keywords:** icEEG, ECoG, cortical network dynamics, distributed cortical networks, ventral temporal cortex, face perception, fusiform face area (FFA), parahippocampal place area (PPA)

## Abstract

Invasive intracranial EEG (icEEG) offers a unique opportunity to study human cognitive networks at an unmatched spatiotemporal resolution. To date, the contributions of icEEG have been limited to the individual-level analyses or cohorts whose data are not integrated in any way. Here we discuss how grouped approaches to icEEG overcome challenges related to sparse-sampling, correct for individual variations in response and provide statistically valid models of brain activity in a population. By the generation of whole-brain activity maps, grouped icEEG enables the study of intra and interregional dynamics between distributed cortical substrates exhibiting task-dependent activity. In this fashion, grouped icEEG analyses can provide significant advances in understanding the mechanisms by which cortical networks give rise to cognitive functions.

## Introduction

The exponential growth in whole-brain neuroimaging studies has produced an overwhelming amount of data, and the conceptual frameworks for the neurobiology of human cognition have undergone tremendous change. These data have produced a consensus that complex cognitive functions—such as language—cannot be understood through the isolated study of specialized, cortical regions (Hagoort, 2014). Currently, a major focus of cognitive neuroscience is to understand how cognition emerges from transient, coordinated neural interactions in distributed large-scale cortical networks (Felleman and Van Essen, 1991; Bressler, 1995; Sporns et al., 2005; Martin, 2007; Patterson et al., 2007; Poeppel, 2014). Driven largely by fMRI, PET, and lesion-based analyses, significant advances have been made in identifying anatomical substrates that form the neural architecture of these distributed networks (Damasio et al., 2004; Dronkers and Ogar, 2004; Binder et al., 2009; Price, 2010; Friederici, 2011; Kanwisher, 2011). However, the limited temporal resolution of these neuroimaging modalities has hindered our understanding of how intra- and interregional cortical interactions give rise to cognition (Lachaux et al., 2003a; Jerbi et al., 2009; Friederici and Singer, 2015).

## Introducing icEEG

A unique opportunity to study cognitive function is presented in patients undergoing intracranial EEG (icEEG) recordings as part of their pre-surgical evaluations for medically refractive focal epilepsy (Mukamel and Fried, 2012). In order to delineate their epileptogenic networks, these patients are implanted with either subdural electrodes that record from the cortical surface or penetrating depth electrodes that record from below the cortical surface, and in some case both (McGonigal et al., 2007; Van Gompel et al., 2008; Tandon et al., 2009). As such, icEEG recordings yield multi-lobar, high spatio-temporal resolution sampling of disseminated brain regions, providing optimal coverage and signal fidelity in comparison to the poor temporal resolution of fMRI/PET and poor spatial resolution of surface EEG/MEG (Jerbi et al., 2009; Lachaux et al., 2012). Importantly, high-frequency broadband gamma activity (BGA, 40–200 Hz) captured by icEEG yields precise estimates of task-related cortical activity, thereby permitting the study of local and long-distance networks at the millisecond time-scales relevant to neural processes (Jacobs and Kahana, 2010; Lachaux et al., 2012).

## The need for grouped icEEG analysis: The sparse-sampling problem

Despite its remarkable advantages, the widespread acceptance of icEEG by cognitive neuroscience has been hindered by difficulties in data representation and analyses at the individual and population-level (for review see Lachaux et al., 2003b; Conner et al., 2013; Kadipasaoglu et al., 2014; Chang, 2015). Relevant to the current discussion are the challenges arising from spatially variable and limited electrode coverage in patients- termed the sparse-sampling problem. This issue is unique to icEEG research, as electrode implantation, and therefore the sites where data are actually collected, are dictated solely by clinical criteria. Therefore, only a fraction of the total brain volume is sampled in any one patient (Halgren et al., 1998), precluding comprehensive investigation of cortical networks at the individual-level.

To address the sparse-sampling problem, and thereby develop icEEG for the study of large-scale, distributed networks, different methods for the grouped analysis of icEEG data have recently been proposed (Miller et al., 2009; Dykstra et al., 2012; Burke et al., 2013; Conner et al., 2013; Davidesco et al., 2013; Kadipasaoglu et al., 2014). Because of the discrete nature of recordings, icEEG activity will likely underestimate functional activity at the individual level. Therefore, a primary goal in all of these methods is to accurately combine data across large numbers of subjects to generate continuous brain activity maps, which leverage the spatiotemporal advantages of icEEG toward providing a more comprehensive view of cortical function. One such method—developed by our lab—employs topologically accurate representations of subdural electrode coverage and BGA on subject-specific cortical models. By integrating this approach with surface-based normalization to precisely align datasets across subjects (Argall et al., 2006; Saad and Reynolds, 2012), and a mixed effects multilevel analysis (MEMA) to correct for unsampled cortical regions (i.e., missing data) (Chen et al., 2011), we are able to perform statistically valid and topologically accurate grouped analyses of icEEG data. In this fashion, our surface-based MEMA (SB-MEMA) can generate continuous brain-activity maps to fully leverage the unique spatio-temporal properties of icEEG in the study of network function (Kadipasaoglu et al., 2014).

To illustrate how such grouped icEEG approaches can contribute to cognitive neuroscience, we discuss SB-MEMA in the context of cortical networks relating to visual object recognition and reading. We note here that the following analyses are intended only as illustrative examples. Therefore, we have not provided detailed experimental methods or statistical interpretations of our results. Furthermore, all results presented in this manuscript are intended solely to highlight the potential application of such grouped icEEG approaches. Importantly, these results will be elaborated in subsequent publications, and this manuscript is not the definitive representation of those analyses.

### Visual object recognition

Visual object recognition is believed to be mediated by neural substrates in the ventral temporal cortex (VTC) capable of categorizing visual inputs within a few 100 ms (Thorpe et al., 1996; Grill-Spector and Kanwisher, 2005). Yet the role of the VTC in accomplishing these complex functional computations remains a mystery. Non-invasive neuroimaging studies have demonstrated a consistent relationship between cortical topology (and white matter connectivity) and functional representations in the VTC (Saygin et al., 2012; Pyles et al., 2013; Grill-Spector and Weiner, 2014; Gomez et al., 2015). Specifically, the mid-fusiform sulcus predicts transitions in the location of cyto-architectonic regions, receptor architectonics, and large-scale functional maps in the VTC (e.g., eccentricity bias/domain-specificity/animacy/real-world object size) (Grill-Spector and Weiner, 2014; Weiner et al., 2014; Gomez et al., 2015). This has led to a hypothesis that the VTC's anatomical organization is spatially optimized for the computational processes of the distinct functional networks subserving object recognition (Grill-Spector and Weiner, 2014). Grouped icEEG studies are uniquely suited to investigate such hypotheses, which require the differentiation of functional networks at millimeter resolution and millisecond time-scales.

**Figure 1:**
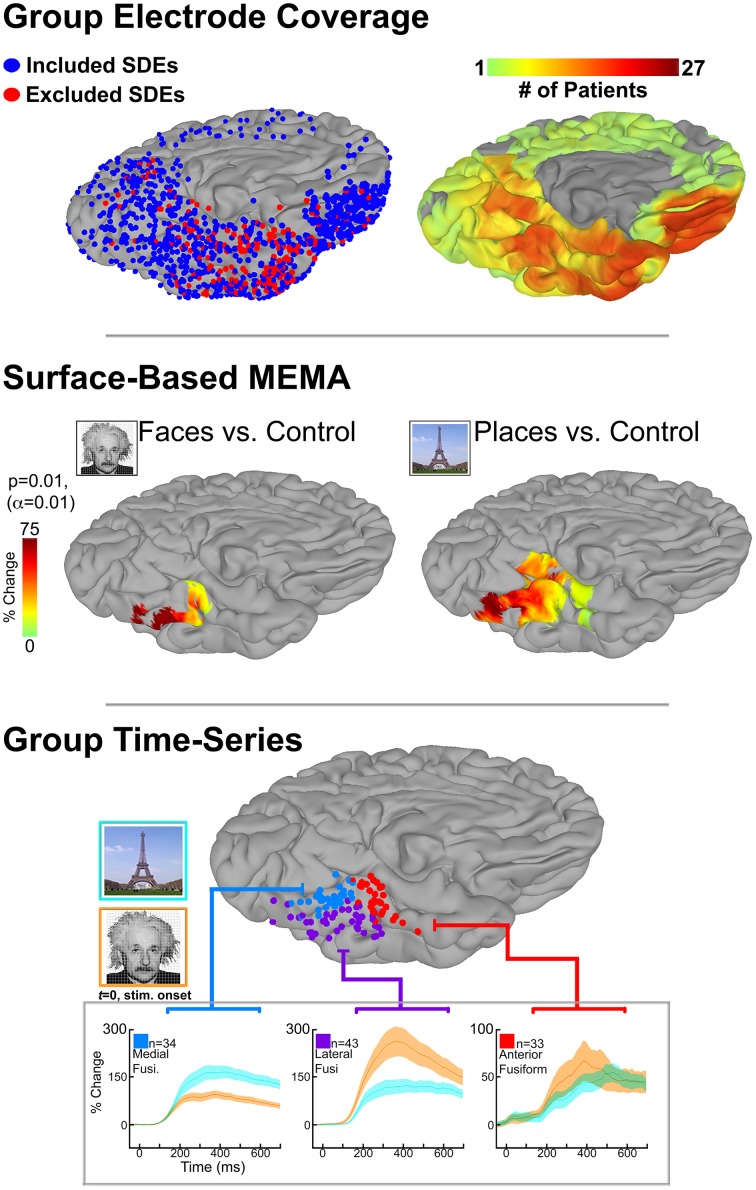
**Top:** icEEG data were collected in 27 patients, implanted with subdural electrodes (SDEs) in the left hemisphere, as they performed a visual confrontation naming of famous faces, places, and scrambled control images. Surface-based representations of SDE coverage and high-frequency broadband gamma activity (BGA; 60–120 Hz) were generated for each subject. We utilize cortical surface models that have been reconstructed from each subject's pre-implantation high-resolution anatomical MRI scans (Phillips Medical; T1-weighted, 1 mm isotropic resolution; using FreeSurfer software), and subsequently imported to the SUMA module of AFNI. Surface-based datasets of SDE coverage and BGA are generated with respect to each subject's cortical model using geodesic metrics to correct for local gyral and sulcal folding patterns. By spatially transforming data to the cortical surface, we integrate SUMA's surface-based normalization strategy to convert individual datasets to a standardized cortical surface (N27). To achieve this, SUMA resamples individual cortical models (and therefore their associated datasets) to a standardized mesh and enables a one-to-one correspondence between anatomical locations across subjects. Group maps for electrode (left) and surface-based coverage (right) are shown for the ventral temporal cortex. SDEs are modeled as spheres, with red spheres indicated SDEs that were excluded due to 60 Hz line noise or epileptiform activity. By grouping data in this fashion, comprehensive cortical coverage is obtained, and cognitive function can be critically evaluated at spatio-temporal scales relevant to neural processes. **Middle:** SB-MEMA derived significant grouped effects estimates by comparing composite BGA percent change (50–500 ms post-stim; with respect to pre-stimulus baseline of −700 to –200 ms) for each stimulus category against its scrambled control. Notably, BGA to faces was localized lateral to the mid-fusiform sulcus, while peak BGA to places was localized medially. Anterior to the mid-fusiform sulcus BGA for both conditions converged in magnitude and spatial extent. **Bottom**: Subject electrodes localized over the three regions in the fusiform (Fusi.) gyrus with significant activity to faces, places, or both stimuli as revealed by SB-MEMA (see B). SDEs are color-coded by region and displayed on a common brain surface (N27). Notably, SDEs are spatially arranged with respect to the mid-fusiform sulcus: laterally (purple), medially (blue), or anteriorly (red). Below, group time-series of percent change in BGA for face (orange) and place (cyan) stimuli can be seen. Of note, traces colored green indicate a region of activity overlap. Percent change is relative to a pre-stimulus baseline (−700 to −200 ms). Stimulus onset at 0 ms. Shading denotes 1 SEM. All figures display the ventro-medial aspect of the left hemisphere (N27 cortical surface model).

To demonstrate this, we use SB-MEMA to investigate category-specific differences in the fusiform gyrus. We applied SB-MEMA to icEEG data collected in a large cohort (*n* = 27, left hemisphere only) as they performed visual confrontation naming of famous faces and places. Importantly, we were able to achieve comprehensive fusiform coverage using the precise inter-subject co-registration afforded by SB-MEMA (Figure 1, top). To focus on early perceptual processes, the analysis was constrained to window from 50 to 500 ms after stimulus presentation. Consistent with previously reported domain-specificity maps, significant BGA for faces and places was localized in a lateral-to-medial fashion, respectively, along the mid-fusiform sulcus (Figure 1, middle; Kanwisher et al., 1997; Epstein and Kanwisher, 1998; Nasr et al., 2011; Grill-Spector and Weiner, 2014).

Given that SB-MEMA computes grouped effects estimates by summing BGA over time, a temporal smoothing of the data is still present. This precludes the evaluation of certain cortical response properties (e.g., onset latency/response duration), which may otherwise provide valuable insight into a given region's functional role (Lachaux et al., 2012). To evaluate the temporal profile of BGA, time-series representations of averaged BGA can be generated from all electrodes contributing to significant loci seen in SB-MEMA (Figure 1, bottom; Conner et al., 2013; Kadipasaoglu et al., 2014). In contrast to summing BGA over a time window, time-series representations instead compute percent change in BGA at each data point (which can be on the order of ms, depending on sampling rate) and plot these changes over time (Yoshor et al., 2007; Kadipasaoglu et al., 2014). Alternatively, data from time-series representations can be spatially transformed back onto the cortical surface to generate 4-dimensional, whole brain representations of cortical activity (Movie 1). Such visualization of time-varying BGA (shown as cortical surface heat maps) relative to cortical anatomy facilitates insights into dynamic network behavior that may not be readily appreciable in static images, and is complementary to SB-MEMA.

Once cortical regions of interest have been identified, more sophisticated measures for assessing functional connectivity and information flow can then be applied to understand how these regions interact during cognitive operations (Bruns et al., 2000; Canolty et al., 2006; Nir et al., 2008; Korzeniewska et al., 2011; Vidal et al., 2012; Watrous et al., 2013; Flinker et al., 2015). To illustrate this, we discuss one such connectivity measure—the short time direct directed transfer function (SdDTF) (Korzeniewska et al., 2008, 2011)—in the context of cortical reading networks. Of note, the electrodes for this example were identified using SB-MEMA for the evaluation of a word-completion task (not shown).

### Network dynamics of reading

**Figure 2:**
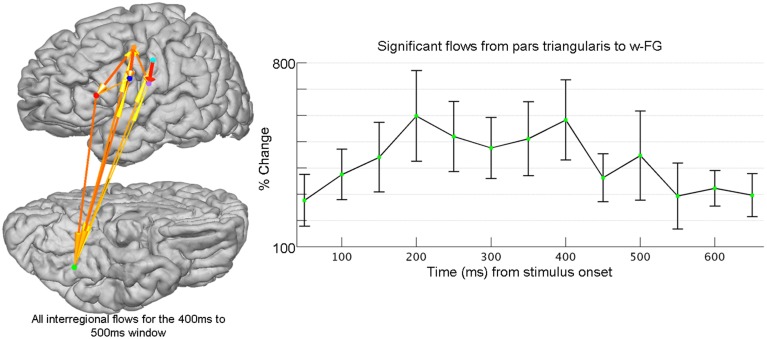
**Frontal-ventral temporal interactions are evaluated using grouped icEEG collected during a word-completion task**. Connectivity is evaluated using the Short-time direct Directed Transfer Function (SdDTF). Post-stimulus interregional flows were determined across post-stimulus windows (100 ms long, 50 ms shift) for high-frequency broadband gamma activity (60–120 Hz) and were compared to pre-stimulus flows computed over one pre-stimulus, baseline window (−700 ms to −200 ms). After normalizing across all patients, all post-stimulus interregional flows were tested for significance (FDR-corrected with a significance level of *p* = 0.05). Shown at right is the time course of percent change of flows (±1 standard error of the mean) from pars triangularis to word-preferential areas in fusiform gyrus (w-FG) that achieved significance. Electrodes for each region (colored spheres) have been identified using SB-MEMA (not shown). The cortical model to the left (lateral view at top, ventral view at bottom; left hemisphere) provides a snapshot of significant flows for the cortical reading network at 400 ms after stimulus onset (w-FG is shown in green and pars triangularis is shown in red). The ability to study long-distance cortical network interactions at millisecond resolution is a unique advantage of grouped icEEG, and enables the critical evaluation of hypotheses regarding functional network dynamics.

The neural substrates that comprise the reading network include cortical areas traditionally associated with language production (e.g., Broca's area), as well a ventrally positioned region in the fusiform gyrus, which demonstrates preferential responses to visually presented words and pseudowords (w-FG) (McCandliss et al., 2003). Cognitive approaches are divided on connectivity patterns during word reading that facilitate the visual processing of orthographic stimuli (Carreiras et al., 2014). While it is agreed upon that w-FG is crucial to word reading, some models predict strictly feed-forward connectivity patterns accompany word reading while other models stress the presence of bi-directional interactions between ventral visual and higher-level frontal cortex (Price and Devlin, 2011; Carreiras et al., 2014). Given that the anatomical sources and temporal evolution of top-down control are not well-established, a data-driven connectivity measure, such as SdDTF, is necessary to investigate the timing and directionality of information transmission during word reading. SdDTF quantifies connectivity across multi-dimensional networks, and can derive directed information flow between any two network nodes, while controlling for the contributions from all other sources (Korzeniewska et al., 2008, 2011). Applied to our icEEG data, patient-specific information flows were computed for subsets of task-relevant electrodes identified through SB-MEMA. It is important to note that connectivity between any two regions can only be derived in patients with electrodes recording simultaneously from both regions. In other words, connectivity measures must first be performed within subject, before individual connectivity estimates can be combined across subjects to yield a grouped connectivity estimate. In this fashion, flows derived from SdDTF were averaged over patient and region, and were able to isolate top-down information flow from Pars Triangularis to w-FG during a word completion task (Figure 2).

## Conclusion

The study of icEEG has been able to generate novel insights into a wide range of cognitive functions (Jacobs and Kahana, 2010; Lachaux et al., 2012). Within the past decade alone, it has significantly advanced diverse areas of neuroscience research, including cognitive control (Wessel et al., 2013), working and episodic memory (Fell et al., 2001; Axmacher et al., 2007; Watrous et al., 2013), sensorimotor integration (Brovelli et al., 2005; Hermes et al., 2011; Bouchard et al., 2013), brain-machine interfaces (Leuthardt et al., 2004, 2011; Miller et al., 2007), perception (Allison et al., 1994, 1999; McCarthy et al., 1999; Privman et al., 2007, 2011; Fisch et al., 2009; Liu et al., 2009; Engell and McCarthy, 2010, 2014; Vidal et al., 2010; Chan et al., 2011; Davidesco et al., 2014; Ghuman et al., 2014), and language processing (Crone et al., 2001; Sahin et al., 2009; Chang et al., 2011; Mesgarani and Chang, 2012; Conner et al., 2013; Flinker et al., 2015). With the development of robust techniques for grouped analysis, icEEG analyses are provided a powerful new tool to investigate the architecture and interregional dynamics of distributed cortical networks. Yet despite its significant advantages, grouped approaches to icEEG still suffer from a number of limitations. Most notably, group-size, and degree of cortical coverage limit the applicability of methods like SB-MEMA. As mentioned earlier, the discrete nature of the recordings may underrepresent functional activity. A failure to find significant effects may be due to the absence of such effects in a given region (true negative) or the lack of sufficient coverage in that region (false negative). Furthermore, as discussed in Section Network Dynamics of Reading, connectivity measures are also dependent on individual with coverage in all regions of interest. For these reasons, it is critical that population-level analyses continue to be supported by data at the individual level.

A final concern that arises with any icEEG study is whether the results found in these patient populations are applicable to the normal human brain. Such concerns are generally addressed using a variety of inclusion criteria, both for patients as well as the data analyzed (e.g., data free of electrophysiological abnormalities, or which arise from pathological cortex) (Halgren et al., 1998; Lachaux et al., 2003b; Crone et al., 2006; Jerbi et al., 2009). The development of grouped icEEG provides a new environment in which to voice these concerns, but also a new opportunity to resolve them. Work from our lab has previously compared patient fMRI and icEEG recordings against fMRI obtained in healthy volunteers, under identical task conditions (Conner et al., 2013). Critically, we identified no significant difference in activity, further validating the reliability of such icEEG recordings. Additionally, work from other groups has begun to investigate the potential of multi-modal analyses by critically investigating grouped icEEG and grouped fMRI analyses from the same patient populations (Mukamel et al., 2005; Privman et al., 2007; He et al., 2008; Conner et al., 2011; Esposito et al., 2012). In doing so, these studies have hoped to better understand the electrophysiological basis of the BOLD signal. Such multi-modal approaches also provide a method for resolving concerns arising from the lack of global coverage in grouped icEEG studies. By integrating data from grouped fMRI and icEEG analyses, it could be confirmed that all relevant components of a given cognitive network have indeed been sampled within the icEEG cohort, prior to subjecting these data to a population-level analysis.

## Author contributions

NT designed research; CK, CC, MR, VB acquired data; CK, MW, KF, CC, VB performed research; CK, KF, MW, CC analyzed data; CK, MW, and NT wrote the paper.

### Conflict of interest statement

The authors declare that the research was conducted in the absence of any commercial or financial relationships that could be construed as a potential conflict of interest.

## Supplementary material

Movie 1 **Intracranial recordings provide unparalleled insights into rapidly evolving patterns of cortical activity across distributed neural substrates**. Electrocorticographic movies of grouped percent change in high-frequency broadband gamma activity (BGA; 60–120 Hz; *n* = 27 subject) during a face and place-naming task are visualized on the N27 cortical surface model. Hotter colors denote an increase in mid-gamma band power, while cooler colors denote a decrease (color-scale ranges from −50 to 50% change). The movie begins 100 ms before stimulus onset and continues until 700 ms after stimulus onset, in 5 ms steps (stimulus onset at 0 ms). Notably, for face stimuli (left surface) increases in BGA are localized to the lateral aspect of the mid-fusiform sulcus. In contrast, place stimuli produce more widespread activations in the medial aspects of the fusiform gyrus.
